# Standardized ileal digestibility of amino acids in cereal grains and co-products in growing pigs

**DOI:** 10.5713/ajas.19.0449

**Published:** 2019-11-12

**Authors:** Su A Lee, Jong Young Ahn, Ah Reum Son, Beob Gyun Kim

**Affiliations:** 1 Department of Animal Science and Technology, Konkuk University, Seoul 05029, Korea; 2 Department of Animal Sciences, University of Illinois at Urbana-Champaign, Urbana, IL 61801, USA; 3 Monogastric Animal Feed Research Institute, Konkuk University, Seoul 05029, Korea

**Keywords:** Alternative Feed Ingredient, Cereal Grains, Co-products, Digestibility, Swine

## Abstract

**Objective:**

The objective was to determine standardized ileal digestibility (SID) of crude protein (CP) and amino acids (AA) in cereal grains and various co-products fed to growing pigs.

**Methods:**

Ten feed ingredients tested were barley (9.3% CP), lupin kernels (31.1% CP), and wheat (11.3% CP) as cereal grains, and 2 sources of corn gluten feed produced in China (21.6% CP) and Korea (24.6% CP), corn gluten meal (65.3% CP), lupin hulls (11.6% CP), rice bran (14.5% CP), soybean meal (44.8% CP), and wheat bran (15.4% CP) as co-products. Ten experimental diets were formulated to contain each ingredient as a sole source of N and an N-free diet was used to correct basal endogenous losses of CP and AA. All diets also contained 0.5% Cr_2_O_3_ as an indigestible index. A replicated 11×6 incomplete Latin square design with 11 dietary treatments, 6 periods, and 22 animals was employed. Twenty-two barrows with an initial body weight of 64.6±4.9 kg were equipped with a T-cannula in the distal ileum. An experimental period consisted of a 4-d adaptation period and a 2-d collection period.

**Results:**

The SID of CP in the barley, lupin kernels, wheat, 2 sources of corn gluten feed, corn gluten meal, lupin hulls, rice bran, soybean meal, and wheat bran were 84.7%, 90.5%, 90.4%, 77.4%, 74.6%, 89.5%, 90.4%, 74.4%, 86.9%, and 63.4% (standard error of the mean [SEM] = 5.3, p = 0.006), respectively. The respective SID values of Lys were 75.5%, 88.4%, 83.9%, 74.7%, 62.4%, 80.3%, 83.9%, 78.5%, 88.0%, and 71.2% (SEM = 3.3, p<0.001), and the SID values of Met were 83.6%, 88.7%, 89.4%, 85.7%, 78.3%, 88.9%, 89.4%, 85.3%, 91.1%, and 77.0% (SEM = 2.4, p<0.001), respectively.

**Conclusion:**

The ileal digestibility of protein and amino acids varies among the feed ingredients fed to pigs.

## INTRODUCTION

Amino acids (AA) are crucial nutrients for the metabolism of pigs. An adequate amount of AA in swine diets based on an accurately determined digestible AA in feed ingredients is important. Ileal digestibility of AA has been measured for an accurate determination of biological availability of AA, and a partial rather than a total collection method using an index has been widely used [1]. The use of standardized ileal digestibility (SID) values enables an accurate formulation due to the additivity [2,3] resulting in maximal N retention and reduced N excretion [4]. The use of SID of AA has been suggested by Stein et al [5] and the AA contents in feed ingredients and the requirement estimates are expressed based on the SID in swine diet formulation [6].

The prices of conventional feed ingredients including corn and soybean meal (SBM) show yearly fluctuation. For an economic advantage, alternative feed ingredients have been used to substitute the conventional feed ingredients [7,8]. Information on the SID of AA in the alternative feed ingredients is necessary to accurately formulate swine diets. However, data on the SID of AA in some feed ingredients including corn gluten feed, lupin hulls, and rice bran are limited in the literature [6]. Thus, the objective of the present study was to determine the SID of crude protein (CP) and AA in the various feed ingredients for pigs.

## MATERIALS AND METHODS

### Animal care

The experimental procedure was approved by the Institutional Animal Care and Use Committee at Konkuk University (KU12127).

### Animals, experimental design, and diets

The ileal digestibility of AA in the feed ingredients was determined by employing a replicated 11×6 incomplete Latin square design with 11 dietary treatments, 6 periods, and 22 animals [9]. Twenty-two barrows with an initial body weight (BW) of 64.6±4.9 kg were equipped with a T-cannula in the distal ileum using the procedures adapted from Stein et al [10], and were individually housed.

Ten feed ingredients were used including barley, lupin ker nels, and wheat as cereal grains, 2 sources of corn gluten feed produced in China (corn gluten feed-C) and Republic of Korea (corn gluten feed-K), corn gluten meal, lupin hulls, rice bran, and wheat bran as co-products, and SBM as a reference ingredient for AA digestibility (Table 1). Eleven experimental diets were prepared (Tables 2, 3). In addition to the 10 diets containing a test feed ingredient as the sole source of AA, an N-free diet was also formulated based on cornstarch and sucrose to determine the basal endogenous losses (BEL) of CP and AA. In all diets, vitamin and mineral premix was also supplemented to meet or exceed the nutrient requirement estimates suggested by NRC [11]. All diets contained 0.5% Cr_2_O_3_ as an indigestible index [1].

### Feeding and sample collection

The daily feed allowance per pig was 2.3 times the metabolizable energy (ME) requirement for maintenance (i.e., 2.3×106 kcal of ME/kg of BW^0.75^) [11] based on a calculated ME of each diet and equally provided twice as meals at 0830 and 1630 h. Water was freely available at all times.

An experimental period consisted of a 4-d adaptation pe riod and a 2-d collection period. The ileal digesta from pigs were collected from 0900 to 1630 h on the collection days by attaching a plastic bag with a wire to the cannula barrel of pigs. Sample bags were changed at least once every 30 min or whenever the bags were filled with digesta. The collected ileal digesta samples were immediately stored at −20°C to prevent bacterial degradation of the AA. At the end of experiment, the samples were freeze-dried and ground before the analyses.

### Chemical analyses

Based on the methods illustrated in AOAC International [12], the feed ingredient, diet, ileal digesta samples were analyzed for dry matter (DM; method 930.15) and CP (method 990.03), and AA concentrations were also analyzed by hydrolyzing with 6 *N* HCl for 24 h at 110°C (method 994.12) except for sulfur-containing AA (method 985.28) and Trp (method 988.15). For Met and Cys, samples were analyzed as methionine sulfone and cysteic acid after cold performic acid oxidation before the acid hydrolysis. For analysis of Trp, samples were hydrolyzed using barium hydroxide. The experimental diets were analyzed for ether extract (method 920.39), crude fiber (method 978.10), ash (method 942.05), Ca (method 978.02), and P (method 946.06). Concentrations of Cr in the diets and ileal digesta samples were analyzed using UV/Vis spectrophotometer (Optizen 2120UV, Mecasys Inc., Deajeon, Korea). Neutral detergent fiber (NDF) and acid detergent fiber (ADF) in the feed ingredient samples were also analyzed using Ankom Technology methods 12 and 13, respectively (Ankom 200 Fiber Analyzer, Ankom Technology, Macedon, NY, USA). Both ADF and NDF were expressed inclusive of residual ash and NDF was assayed with a heat stable amylase.

### Calculations

The apparent ileal digestibility (AID) and SID of CP and AA were calculated based on CP, AA, and Cr concentrations in diets and ileal digesta. Because each feed ingredient was the sole source of N in the experimental diets, the AID or SID of a diet was considered as the AID or SID in each feed ingredient. The AID of CP and AA were calculated based on the following equation [1]:

$$\text{AID }(\%)=[1-({\text{Cr}}_{\text{input}}/{\text{Cr}}_{\text{output}})\times ({\text{AA}}_{\text{output}}/{\text{AA}}_{\text{input}})]\times 100$$

where Cr_input_ and Cr_output_ represented the Cr concentrations (g/kg) in the diets and ileal digesta from pigs, respectively; AA_input_ and AA_output_ represented the CP or AA concentrations (g/kg) in the diets and ileal digesta from the pigs, respectively.

The BEL of CP and AA (g/kg of DM intake) were based on the following equation [1]:

$$\text{BEL of CP and AA }(\text{g}/\text{kg of DM intake})=({\text{Cr}}_{\text{input}}/{\text{Cr}}_{\text{output}})\times {\text{AA}}_{\text{output}}$$

where Cr_input_ and Cr_output_ represented the Cr concentrations (g/kg of DM) in an N-free diet and ileal digesta from pigs fed the N-free diet, respectively; AA_output_ represented the CP or AA concentrations (g/kg of DM) in the ileal digesta from pigs fed the N-free diet.

The SID of CP and AA were calculated based on the fol lowing equation [1]:

$$\text{SID }(\%)=\text{AID }(\%)+[100\times (\text{BEL}/{\text{AA}}_{\text{input}})]$$

### Statistical analysis

Data were analyzed using the MIXED procedure of SAS (SAS Inst. Inc., Cary, NC, USA). An initial model included diet as a fixed effect and replication, period nested within replication, and animal nested within replication as random effects. The random variables were not significant and consequently were excluded from the final model. Least squares mean separation in the AID and SID of CP and AA among the feed ingredients was performed using the PDIFF option with Tukey’s adjustment. The pig was the experimental unit, and significance was determined at p-values less than 0.05.

## RESULTS

### Nutrient composition

The CP concentration in the feed ingredients used in the present study ranged from 9.3% to 65.3% (Table 1). Lysine:CP ratios that generally represent the quality of proteins in wheat and corn co-products were less than those in other feed ingredients. Analyzed CP and AA concentrations in the experimental diets except the lupin hull-containing diet agreed with the calculated values (Table 3).

### Apparent ileal digestibility of crude protein and amino acids

The AID of CP and AA in the 10 feed ingredients differed from each other (p<0.05; Table 4). The AID of CP and indispensable AA in lupin kernels were greater (p<0.05) than those in barley and wheat except Arg, Lys, Met, and Trp. The AID of CP and AA in corn gluten meal and SBM did not differ from each other. The AID of Arg, Lys, and Trp in the 2 sources of corn gluten feeds were less (p<0.05) compared with the values in SBM, and the AID of Ile, Leu, Phe, Thr, and Val in rice bran were also less (p<0.05) than the values in SBM. The AID values for CP and most AA in lupin hulls and wheat bran were less (p<0.05) than those in SBM.

### Standardized ileal digestibility of crude protein and amino acids

The SID of CP and AA in the 11 feed ingredients differed from each other (p<0.05; Table 5) except for Arg and Gly. The SID of CP and most AA in corn gluten feed-C, corn gluten meal, lupin hulls did not differ. The SID of Ile, Leu, Phe, and Val in rice bran were less (p<0.05) than other co-products except for wheat bran. The SID values of most indispensable AA in wheat bran were less than the values in the SBM (p<0.05).

## DISCUSSION

The CP and most indispensable AA in barley, lupin kernels, corn gluten feed-C, SBM, and wheat bran agreed with the previous values [6,13,14]. The CP and most AA concentrations of wheat and rice bran used in the present study were less than those reported previously [6,14,15], but similar to those in Sauvant et al [13]. The CP and AA concentrations in corn gluten feed-K and corn gluten meal were greater than the values presented by the literature [6,13,14]. The AA concentrations in lupin hulls used in this study were about twice greater compared to the data reported by Fernández and Batterham [16]. The reason for these discrepancies in the nutrient compositions of co-products among studies may be explained by several factors including different variety, growing environment, and processing methods and conditions [14]. Information on the AA compositions or digestibility of lupin hulls was not available to be compared.

The BEL of CP and AA determined from the pigs fed the N-free diet agreed with the previous studies [7,17] and the predicted BEL using an equation suggested by Park et al [18]. The AID of Pro in some feed ingredients showed negative values if an experimental diet contained low Pro concentration, which may be associated with low Pro digestibility, resulting in a larger contribution from the BEL of Pro that originated from mostly mucin and enzyme secretions to the ileal digesta [19,20]. The observation that the BEL of Pro was the greatest among other AA resulted also in large difference between the AID and SID of Pro in the feed ingredients.

The SID of most AA and CP in feed ingredients tested in this study were within a range of data in the literature [6,13, 14]. The SID of CP and AA in SBM used in the present study were similar to the values presented in the reviews of the literature [6,13,14] and the recent publications [21,22]. The SID of most AA and CP in barley [23,24] and wheat [25,26] were within a range of data in recent publications. Almeida et al [27] reported the SID values of AA in the co-products of corn fed to pigs, and the SID values were similar compared to the values in the corn gluten feed-C and-K in the present study. The SID of CP and AA in corn gluten meal used in the present study were within a range of data in the literature [27,28]. While corn gluten feed and corn gluten meal were the co-products from corn, the SID values were variable between the sources, which may be explained by different concentrations of corn fractions [29]. The SID of CP and AA in lupin hulls was similar to that in SBM. To our knowledge, there have been no data on the SID of AA in lupin hulls reported previously. For rice bran, the SID of CP and AA observed in the present study was less than the values reported by Casas et al [15], but greater than the values by Huang et al [30]. There were variations in the CP and AA contents in the full fat rice bran used among the experiments, but the SID values are not be affected by the total contents of CP and AA [5]. The greater fat contents in rice bran may increase the greater AA digestibility in pigs [31,32], but the crude fat concentrations in the rice bran were similar among the sources used in the present study and the previous studies. The difference in fiber contents may also affect the AA digestibility in rice bran. It is possible that dietary NDF can decrease the digestibility of CP [33], and the NDF contents in rice bran were 21.4% DM in the present study (Table 1) whereas the NDF in rice bran was 23.8% DM [30] and 5.1% DM [15], respectively. The SID in wheat bran used in this study was in agreement with the values reported by Eklund et al [25].

## CONCLUSION

The values for the protein and AA digestibility in various feed ingredients in this study will be useful for the accurate formulation of swine diets. The ileal digestibility of protein and AAs varies among the feed ingredients fed to pigs.

## Figures and Tables

**Table 1:** Analyzed nutrient composition in feed ingredients (as-fed basis)

Item (%)	Cereal grains			Co-products						
	
	Barley	Lupin kernels	Wheat	Corn gluten feed-China	Corn gluten feed-Korea	Corn gluten meal	Lupin Hulls	Rice bran	Soybean meal	Wheat bran
Dry matter	91.18	91.53	92.75	90.94	88.56	90.85	91.82	87.54	90.09	90.35
Crude protein	9.32	31.13	11.30	21.56	24.55	65.25	11.58	14.51	44.80	15.43
Crude fiber	4.35	6.46	2.44	9.93	7.23	1.43	39.56	6.14	4.82	7.68
Neutral detergent fiber	16.19	11.54	14.22	38.38	29.52	4.97	57.37	18.73	11.88	37.07
Acid detergent fiber	6.63	9.48	3.57	12.35	9.87	3.77	49.17	9.78	6.51	10.87
Lys:crude protein	4.40	5.82	3.36	3.38	3.37	1.81	5.95	5.66	6.88	4.42
Indispensable amino acids										
Arg	0.51	3.84	0.60	0.93	1.24	2.17	0.82	1.18	3.44	1.03
His	0.22	0.99	0.29	0.64	0.77	1.37	0.29	0.43	1.28	0.43
Ile	0.31	1.45	0.42	0.73	0.77	2.69	0.44	0.48	2.17	0.48
Leu	0.66	2.50	0.81	1.97	2.55	11.8	0.76	1.00	3.81	0.99
Lys	0.41	1.81	0.38	0.73	0.83	1.18	0.69	0.82	3.08	0.68
Met	0.16	0.21	0.20	0.25	0.42	1.60	0.11	0.25	0.65	0.22
Phe	0.48	1.44	0.54	0.87	1.05	4.40	0.47	0.64	2.62	0.65
Thr	0.35	1.37	0.36	0.78	0.90	2.41	0.44	0.57	1.98	0.53
Trp	0.09	0.25	0.08	0.07	0.06	0.24	0.07	0.12	0.48	0.15
Val	0.46	1.48	0.53	1.04	1.17	3.10	0.50	0.73	2.25	0.68
Dispensable amino acids										
Ala	0.42	1.32	0.46	1.61	1.73	6.04	0.46	0.89	2.14	0.75
Asp	0.63	3.55	0.63	1.19	1.60	4.17	1.01	1.38	5.44	1.06
Cys	0.27	0.56	0.35	0.56	0.71	1.47	0.18	0.37	0.88	0.40
Glu	2.03	7.68	3.25	3.23	4.21	15.4	1.84	1.97	8.88	3.02
Gly	0.41	1.56	0.49	0.94	1.06	1.81	0.49	0.78	2.03	0.82
Pro	0.79	1.09	0.93	1.66	2.10	5.77	0.37	0.54	1.98	0.85
Ser	0.41	1.73	0.51	0.85	1.08	3.65	0.52	0.65	2.46	0.66
Tyr	0.29	1.20	0.31	0.59	0.83	3.53	0.32	0.43	1.71	0.43

**Table 2:** Ingredient composition of experimental diets (as-fed basis)

Item (%)	Diet										

	Cereal grains			Co-products							N-free
	
	Barley	Lupin kernels	Wheat	Corn gluten feed-China	Corn gluten feed-Korea	Corn gluten meal	Lupin hulls	Rice bran	Soybean meal	Wheat bran	
Corn starch	27.15	37.20	27.15	37.35	37.20	50.50	37.50	27.40	44.15	27.45	68.35
Sucrose	20.00	20.00	20.00	20.00	20.00	20.00	20.00	20.00	20.00	20.00	20.00
Barley	50.00	-	-	-	-	-	-	-	-	-	-
Lupin kernels	-	40.00	-	-	-	-	-	-	-	-	-
Wheat	-	-	50.00	-	-	-	-	-	-	-	-
Corn gluten feed-China	-	-	-	40.00	-	-	-	-	-	-	-
Corn gluten feed-Korea	-	-	-	-	40.00	-	-	-	-	-	-
Corn gluten meal	-	-	-	-	-	26.50	-	-	-	-	-
Lupin hulls	-	-	-	-	-	-	40.00	-	-	-	-
Rice bran	-	-	-	-	-	-	-	50.00	-	-	-
Soybean meal	-	-	-	-	-	-	-	-	33.00	-	-
Wheat bran	-	-	-	-	-	-	-	-	-	50.00	-
Soybean oil	-	-	-	-	-	-	-	-	-	-	4.00
Cellulose	-	-	-	-	-	-	-	-	-	-	4.00
K_2_CO_3_	-	-	-	-	-	-	-	-	-	-	0.40
MgO	-	-	-	-	-	-	-	-	-	-	0.10
Ground limestone	0.75	0.80	0.85	0.70	1.00	0.90	0.10	1.20	1.00	1.15	0.75
Dicalcium phosphate	0.70	0.60	0.60	0.55	0.40	0.70	1.00	-	0.45	-	1.00
Salt	0.40	0.40	0.40	0.40	0.40	0.40	0.40	0.40	0.40	0.40	0.40
Vitamin-mineral premix1)	0.50	0.50	0.50	0.50	0.50	0.50	0.50	0.50	0.50	0.50	0.50
Cr_2_O_3_	0.50	0.50	0.50	0.50	0.50	0.50	0.50	0.50	0.50	0.50	0.50

1) Provided the following quantities per kg of a diet: vitamin A, 25,000 IU; vitamin D_3_, 4,000 IU; vitamin E, 50 IU; vitamin K, 5.0 mg; thiamin, 4.9 mg; riboflavin, 10.0 mg; pyridoxine, 4.9 mg; vitamin B_12_, 0.06 mg; pantothenic acid, 37.5 mg; folic acid, 1.10 mg; niacin, 62 mg; biotin, 0.06 mg; Cu, 25 mg as CuSO_4_; Fe, 268 mg as FeSO_4_; I, 5.0 mg as KIO_3_; Mn, 125 mg as MnSO_4_; Se, 0.38 mg as Na_2_SeO_3_; Zn, 313 mg as ZnO; butylated hydroxytoluene, 50 mg.

**Table 3:** Analyzed nutrient composition of experimental diets (as-fed basis)

Item (%)	Diet										

	Cereal grains			Co-products							N-free
	
	Barley	Lupin kernels	Wheat	Corn gluten feed-China	Corn gluten feed-Korea	Corn gluten meal	Lupin hulls	Rice bran	Soybean meal	Wheat bran	
Dry matter	91.35	91.67	92.48	92.23	91.48	91.41	91.51	91.03	91.10	90.62	91.99
Crude protein	5.22	12.17	5.97	8.66	10.83	19.89	3.94	8.19	15.49	7.96	0.48
Ether extract	0.79	2.05	0.73	0.58	1.45	0.15	0.65	9.09	0.50	1.56	2.74
Crude fiber	2.47	3.71	1.52	3.98	2.83	0.80	14.6	3.09	2.94	4.35	2.03
Ash	3.68	3.65	3.53	4.55	5.84	3.06	3.35	6.60	4.80	4.69	3.53
Ca	0.51	0.61	0.51	0.47	0.66	0.58	0.50	0.59	0.76	0.53	0.60
P	0.25	0.29	0.26	0.35	0.45	0.21	0.27	0.90	0.33	0.45	0.20
Indispensable amino acids											
Arg	0.22	1.36	0.30	0.37	0.43	0.53	0.24	0.53	1.09	0.54	0.01
His	0.10	0.36	0.14	0.26	0.31	0.38	0.08	0.20	0.41	0.23	-
Ile	0.14	0.50	0.20	0.27	0.35	0.73	0.12	0.22	0.69	0.26	0.01
Leu	0.29	0.91	0.40	0.78	0.99	3.19	0.22	0.45	1.21	0.52	0.02
Lys	0.18	0.63	0.20	0.30	0.32	0.33	0.21	0.39	0.97	0.38	0.01
Met	0.07	0.11	0.11	0.12	0.13	0.40	0.05	0.14	0.21	0.13	-
Phe	0.22	0.50	0.27	0.35	0.43	1.17	0.14	0.31	0.78	0.35	0.01
Thr	0.16	0.49	0.19	0.33	0.34	0.65	0.14	0.27	0.64	0.29	0.01
Trp	0.03	0.11	0.06	0.03	0.02	0.07	0.01	0.07	0.14	0.05	-
Val	0.21	0.50	0.27	0.43	0.45	0.82	0.17	0.33	0.74	0.38	0.02
Dispensable amino acids											
Ala	0.19	0.45	0.24	0.65	0.65	1.62	0.15	0.41	0.70	0.41	0.01
Asp	0.29	1.25	0.33	0.50	0.60	1.13	0.30	0.65	1.77	0.58	0.02
Cys	0.11	0.25	0.17	0.23	0.23	0.37	0.09	0.20	0.28	0.21	-
Glu	0.96	2.68	1.68	1.36	1.58	4.11	0.56	0.92	2.92	1.65	0.05
Gly	0.19	0.57	0.25	0.39	0.40	0.50	0.15	0.36	0.67	0.44	0.01
Pro	0.37	0.24	0.50	0.71	0.79	1.58	0.12	0.25	0.69	0.47	0.01
Ser	0.19	0.61	0.28	0.36	0.40	0.96	0.18	0.30	0.79	0.36	0.02
Tyr	0.11	0.41	0.14	0.20	0.26	0.68	0.10	0.19	0.46	0.22	-

**Table 4:** Apparent ileal digestibility of crude protein and amino acids in feed ingredients

Item (%)	Cereal grains			Co-products							SEM	p-value
	
	Barley	Lupin kernels	Wheat	Corn gluten feed-China	Corn gluten feed- Korea	Corn gluten meal	Lupin hulls	Rice bran	Soybean meal	Wheat bran		
Observation (n)	9	11	8	9	7	8	8	5	9	8	-	-
CP	51.5^c^	75.9^ab^	49.9^c^	55.3^c^	59.3^bc^	80.5^a^	49.9^c^	59.9abc	74.5^ab^	43.4^c^	4.3	<0.001
Indispensable AA												
Arg	60.4^c^	92.7^a^	79.1^ab^	71.9^bc^	70.0^bc^	79.9^ab^	79.1^ab^	83.4^ab^	89.6^a^	81.8^ab^	3.3	<0.001
His	68.3^cd^	86.8^a^	66.4^d^	78.2^ab^	76.2bcd	84.4^ab^	66.4^d^	76.9abcd	86.4^ab^	78.3abc	2.3	<0.001
Ile	53.6^cd^	83.2^a^	59.6^cd^	67.6^bc^	69.5abc	82.3^ab^	59.6^cd^	45.4^d^	81.0^ab^	60.2^cd^	3.8	<0.001
Leu	64.9^cd^	85.3^a^	65.7^cd^	82.0^ab^	79.1^ab^	87.8^a^	65.7^cd^	58.6^d^	82.9^a^	66.7^cd^	2.5	<0.001
Lys	59.3^bc^	83.7^a^	71.0^ab^	64.1^bc^	53.7^c^	71.2^ab^	71.0^ab^	73.2^ab^	84.7^a^	64.0^bc^	3.5	<0.001
Met	74.0^bc^	82.4abc	77.0abc	79.5abc	73.3^bc^	87.1^a^	77.0abc	82.1abc	87.5^a^	72.2^c^	2.7	<0.001
Phe	70.8cde	84.1^ab^	64.6^de^	77.9abc	73.7bcd	86.1^a^	64.6^de^	60.3^e^	83.1^ab^	69.0cde	2.4	< 0.001
Thr	45.3^c^	77.5^a^	47.4^c^	59.3^bc^	52.4^c^	77.8^a^	47.4^c^	47.4^c^	76.4^ab^	47.0^c^	4.1	<0.001
Trp	56.4abc	84.7^a^	24.3^cd^	43.5bcd	6.06^d^	75.4^ab^	24.3^cd^	63.4abc	83.2^a^	39.5bcd	8.2	<0.001
Val	52.0^d^	75.7^ab^	52.7^d^	69.5abc	61.4bcd	79.8^a^	52.7^d^	51.5^d^	77.2^a^	54.8^d^	3.3	<0.001
Dispensable AA												
Ala	43.9^c^	72.3^ab^	47.7^c^	75.8^ab^	69.6^ab^	84.2^a^	47.7^c^	58.0^bc^	74.1^ab^	48.5^c^	4.1	<0.001
Asp	50.5^c^	83.0^a^	61.7^c^	60.4^c^	56.3^c^	79.2^ab^	61.7^c^	63.6^bc^	82.6^a^	57.4^c^	3.4	<0.001
Cys	60.3^cd^	82.9^a^	70.5abcd	62.0bcd	54.8^d^	77.1^ab^	70.5abcd	65.7abcd	74.6abc	62.8bcd	3.6	<0.001
Glu	79.0^cd^	89.5^a^	75.4^d^	77.7^d^	75.4^d^	86.7abc	75.4^d^	74.4^d^	86.4abc	81.1bcd	1.8	<0.001
Gly	0.87^d^	71.4^a^	11.5^cd^	32.7bcd	32.6bcd	58.1^ab^	11.5^cd^	40.8abcd	60.7^ab^	46.5abc	8.7	<0.001
Pro	−78.8^ab^	−59.1^ab^	−177^b^	−23.5^ab^	7.78^ab^	55.9^a^	−177^b^	−124^ab^	0.14^ab^	−28.7^ab^	44.7	0.032
Ser	53.9^c^	81.8^a^	57.8^bc^	65.5^bc^	63.6^bc^	83.0^a^	57.8^bc^	55.9^bc^	81.6^a^	62.2^bc^	2.9	<0.001
Tyr	58.6^d^	85.3^a^	66.0^d^	68.1^cd^	67.7bcd	82.5^ab^	66.0^d^	61.7^d^	80.7abc	64.7^d^	3.1	<0.001

SEM, standard error of the mean; CP, crude protein; AA, amino acids.

a–e Within a row, means without a common superscript differ (p<0.05).

**Table 5:** Standardized ileal digestibility of crude protein and amino acids in feed ingredients1)

Item (%)	Cereal grains			Co-products							SEM	p-value
	
	Barley	Lupin kernels	Wheat	Corn gluten feed-China	Corn gluten feed-Korea	Corn gluten meal	Lupin hulls	Rice bran	Soybean meal	Wheat bran		
Observation (n)	9	11	8	9	7	8	8	5	9	8	-	-
CP	84.7^ab^	90.5^a^	90.4^a^	77.4^ab^	74.6^ab^	89.5^a^	90.4^a^	74.4^ab^	86.9^ab^	63.4^b^	5.3	0.006
Indispensable AA												
Arg	86.9	97.2	99.8	90.6	82.5	91.7	99.8	88.1	95.9	91.0	4.5	0.238
His	83.0	91.0	83.5	84.3	80.8	88.4	83.5	82.3	90.3	84.2	2.4	0.016
Ile	77.0abc	89.8^a^	86.3^ab^	80.1^ab^	78.8abc	86.8^ab^	86.3^ab^	59.0^c^	85.9^ab^	72.5^bc^	3.9	<0.001
Leu	80.0abc	90.2^a^	85.1^ab^	87.8^a^	83.4^ab^	89.2^a^	85.1^ab^	67.3^c^	86.7^a^	74.9^bc^	2.6	<0.001
Lys	75.5abc	88.4^a^	83.9^ab^	74.7abc	62.4^c^	80.3^ab^	83.9^ab^	78.5abc	88.0^a^	71.2^bc^	3.3	<0.001
Met	83.6abc	88.7^ab^	89.4^ab^	85.7abc	78.3^bc^	88.9^ab^	89.4^ab^	85.3abc	91.1^a^	77.0^c^	2.4	<0.001
Phe	83.2^ab^	89.6^a^	83.8^ab^	85.9^ab^	79.9abc	88.5^a^	83.8^ab^	68.4^c^	86.7^ab^	76.7^bc^	2.4	<0.001
Thr	76.3abc	87.7^a^	81.7abc	75.0abc	66.7^bc^	85.5^ab^	81.7abc	63.5^bc^	84.4^ab^	63.6^c^	4.3	<0.001
Trp	80.6^ab^	91.5^a^	92.8^a^	69.6abc	41.2^c^	86.1^ab^	92.8^a^	71.4abc	88.8^ab^	53.2^bc^	8.5	<0.001
Val	77.4abc	86.5^a^	82.7abc	82.5^ab^	72.9abc	86.5^a^	82.7abc	65.0^c^	84.8^a^	68.2^bc^	3.4	<0.001
Dispensable AA												
Ala	75.2^ab^	85.8^a^	84.6^a^	85.8^a^	78.4^ab^	88.0^a^	84.6^a^	68.5^ab^	83.3^a^	62.0^b^	4.5	<0.001
Asp	73.4^bc^	88.4^a^	82.6abc	74.6abc	67.0^c^	85.2^ab^	82.6abc	71.6abc	86.6^ab^	68.2^c^	3.6	<0.001
Cys	77.9abcd	90.9^a^	90.4^a^	71.3^cd^	62.9^d^	82.5abc	90.4^a^	72.5bcd	82.2abc	71.3^cd^	3.3	<0.001
Glu	87.4abc	92.5^a^	88.9abc	84.1^bc^	80.3^c^	88.7abc	88.9abc	81.0^bc^	89.4^ab^	85.7abc	1.9	<0.001
Gly	75.0	97.0	95.5	73.9	65.6	87.8	95.5	63.4	84.7	75.2	12.1	0.529
Pro	106^ab^	237^ab^	328^a^	87.3^b^	88.6^ab^	102^ab^	328^a^	24.9^b^	114^ab^	100^ab^	51.9	0.007
Ser	80.2abc	90.2^a^	83.9abc	80.4abc	75.7abc	88.4^ab^	83.9abc	68.7^c^	88.4^ab^	75.3^bc^	3.3	<0.001
Tyr	78.9^ab^	90.8^a^	87.8^ab^	79.6^ab^	76.1^b^	85.8^ab^	87.8^ab^	72.4^b^	85.6^ab^	74.6^b^	3.0	<0.001

SEM, standard error of the mean; CP, crude protein; AA, amino acids; SID, standardized ileal digestibility.

1) Each SID of CP and AA was calculated by correcting apparent ileal digestibility of CP and AA for basal endogenous losses. Basal endogenous losses (g/kg dry matter intake) were determined based on the Cr, CP, and AA concentrations in the N-free diet and the ileal digesta from pigs fed the N-free diet as: CP, 22.8; Arg, 0.87; His, 0.19; Ile, 0.38; Leu, 0.51; Lys, 0.38; Met, 0.09; Phe, 0.31; Thr, 0.55; Trp, 0.09; Val, 0.64; Ala, 0.76; Asp, 0.83; Cys, 0.25; Glu, 1.01; Gly, 1.96; Pro, 9.68; Ser, 0.63; Tyr, 0.26.

a–d Within a row, means without a common superscript differ (p<0.05).
